# Supplementary material for: Levels of heavy metals in soil and vegetables and associated health risks in Mojo area, Ethiopia
Source: PLoS One. 2020 Jan 30;15(1):e0227883. doi: 10.1371/journal.pone.0227883 (PMC6992214; doi:10.1371/journal.pone.0227883)
Supplement: S1 Table — (PDF) [file pone.0227883.s001.pdf]

**S1 Table** Optimal conditions achieved for soil and vegetables samples digestion procedures.

| No. | Reagent volumes (mL) | | | Temperature(°C) | Pressure (w) | Time (min) | Result /Observation |
|---|---|---|---|---|---|---|---|
| | $HNO_3$ | HCl | Total | | | | |
| 1 | 4mL | 8mL | 12mL | 70-130 | 25-55 | 20 | Deep yellow |
| 2 | 5mL | 7mL | 12mL | 80-140 | 30-60 | 25 | Deep yellow |
| 3 | 6mL | 6mL | 12mL | 90-150 | 35-65 | 30 | Deep yellow |
| 4 | 7mL | 5mL | 12mL | 100-160 | 40-70 | 35 | clear and pale |
| 5 | 8mL | 4mL | 12mL | 110-170 | 45-75 | 40 | Clear and pale |
| 6 | ***9mL** | ***3mL** | **12mL** | ***120-180** | ***50-80** | ***45** | ***Clear and Colorless** |
| 7 | 10mL | 2mL | 12mL | 130-190 | 55-85 | 50 | Clear with suspension |
| 8 | 11mL | 1mL | 12mL | 140-200 | 60-90 | 55 | Clear with suspension |

*Indicates that the optimal condition for soil, vegetables and water samples digestion procedure.
