# Supplementary material for: Levels of heavy metals in soil and vegetables and associated health risks in Mojo area, Ethiopia
Source: PLoS One. 2020 Jan 30;15(1):e0227883. doi: 10.1371/journal.pone.0227883 (PMC6992214; doi:10.1371/journal.pone.0227883)
Supplement: S2 Table — (PDF) [file pone.0227883.s002.pdf]

S2 Table Instrumental operating condition for the analysis of metal in soil and vegetable samples.

Parameters	Value
Plasma power	1400W
Pump speed	30 rpm
Coolant flow	13 L/min
Auxiliary flow	0.8 L/min
Nebulizer flow	0.73 L/min
Optical temperature	14.0-16.0 °C
Nebulizer pressure	2.0-4.0 Bar
Main Argon Pressure	6.0-8.0 Bar
Replicates	3
