# Supplementary material for: Levels of heavy metals in soil and vegetables and associated health risks in Mojo area, Ethiopia
Source: PLoS One. 2020 Jan 30;15(1):e0227883. doi: 10.1371/journal.pone.0227883 (PMC6992214; doi:10.1371/journal.pone.0227883)
Supplement: S3 Table — (PDF) [file pone.0227883.s003.pdf]

S3 Table Method detection limits (MDL) and limit of quantification (LOQ) for vegetable and soil samples analysis.

Heavy metals	IDL (mg/L)	Vegetable samples		Soil Samples	
		MDL (mg/L)	LOQ (mg/L)	MDL (mg/L)	LOQ (mg/L)
As	0.0001	0.0011	0.084	0.0016	0.185
Pb	0.0004	0.0007	0.057	0.0006	0.034
Cd	0.0003	0.0005	0.063	0.0008	0.035
Zn	0.0004	0.0007	0.084	0.0012	0.746
Cu	0.0006	0.0013	0.047	0.0015	0.043
Fe	0.0001	0.0004	0.036	0.0008	0.066
Mn	0.0001	0.0002	0.069	0.0004	0.070
Cr	0.0007	0.0014	0.033	0.0016	0.032
Hg	0.0006	0.0015	0.023	0.0017	0.021
Ni	0.0004	0.0011	0.018	0.0015	0.016
Co	0.0002	0.0013	0.024	0.0019	0.011
