# Supplementary material for: Levels of heavy metals in soil and vegetables and associated health risks in Mojo area, Ethiopia
Source: PLoS One. 2020 Jan 30;15(1):e0227883. doi: 10.1371/journal.pone.0227883 (PMC6992214; doi:10.1371/journal.pone.0227883)
Supplement: S4 Table — (PDF) [file pone.0227883.s004.pdf]

S4 Table Percentage recovery data of the method used for soil sample digestion (M±SD, n = 3)

Heavy Metal	Concentration before spiking (M± SDs) (mg/kg)	Amount spiked (mg/kg)	Concentration after spiking (M± SD) (mg/kg)	% Recovery	% RSD
Cr	0.414±0.01	5	6.04±0.014	112.52	2.42
Cd	0.06±0.012	5	5.86±0.013	116.00	20.0
Zn	1.069±0.035	5	6.55±0.015	109.62	3.27
Fe	0.453±0.053	5	6.04±0.023	111.74	11.69
Pb	0.430±0.021	5	5.93±0.227	110.00	4.88
Cu	0.263±0.001	5	5.76±0.011	109.94	0.38
As	0.324 ±0.059	5	5.92±0.233	111.92	8.21
Mn	0.177±0.005	5	5.65±0.006	109.46	8.48
Hg	0.165±0.008	5	5.26±0.017	101.9	4.84
Ni	0.35±0.008	5	5.47±0.014	109.4	2.28
Co	0.136±0.027	5	5.76±0.014	112.08	9.85
