# Supplementary material for: Levels of heavy metals in soil and vegetables and associated health risks in Mojo area, Ethiopia
Source: PLoS One. 2020 Jan 30;15(1):e0227883. doi: 10.1371/journal.pone.0227883 (PMC6992214; doi:10.1371/journal.pone.0227883)
Supplement: S5 Table — (PDF) [file pone.0227883.s005.pdf]

S5 Table Percentage recovery values of the method used for tomato digestion (M±SD, n = 3)

Heavy Metal	Concentration before spiking (M± SD) (ppm)	Amount spiked (ppm)	Concentration after spiking (M± SD) (ppm)	% Recovery	% RSD
Cr	0.12 ± 0.013	5	5.78±0.018	113.20	10.83
Cd	0.124±0.004	5	5.85±0.026	114.52	3.22
Zn	0.222±0.007	5	5.87±0.02	112.96	3.15
Fe	0.432±0.002	5	6.23±0.022	115.96	0.46
Pb	0.024±0.004	5	5.68±0.151	113.12	6.66
Cu	0.134±0.006	5	5.54±0.015	108.12	4.48
As	0.008± 0.001	5	5.8±0.132	115.84	8.50
Mn	0.277±0.004	5	6.15±0.004	117.46	1.44
Hg	0.032±0.001	5	5.33±0.001	105.96	3.12
Ni	0.009±0.001	5	5.28±0.021	105.42	11.11
Co	0.004±0.0001	5	5.23±0.016	104.52	2.5
