# Supplementary material for: Levels of heavy metals in soil and vegetables and associated health risks in Mojo area, Ethiopia
Source: PLoS One. 2020 Jan 30;15(1):e0227883. doi: 10.1371/journal.pone.0227883 (PMC6992214; doi:10.1371/journal.pone.0227883)
Supplement: S6 Table — (PDF) [file pone.0227883.s006.pdf]

S6 Table Percentage recovery values of the method used for cabbage digestion (M±SD, n = 3)

Heavy Metal	Concentration before spiking (M± SD) (ppm)	Amount spiked (ppm)	Concentration after spiking (M± SD) (ppm)	% Recovery	% RSD
Cr	0.390±0.03	5	5.10±0.02	94.20	7.69
Cd	0.230±0.004	5	5.40±0.01	103.40	1.74
Zn	0.354±0.01	5	5.73±0.02	107.52	2.82
Fe	0.330±0.06	5	5.03±0.17	94.00	8.18
Pb	0.262± 0.05	5	5.45±0.21	103.76	9.08
Cu	0.072±0.01	5	5.07±0.02	99.96	10.88
As	0.059±0.01	5	5.36±0.20	106.02	6.95
Mn	0.076±0.01	5	5.18±0.02	102.08	10.16
Hg	0.042±0.003	5	5.21±0.01	103.36	7.14
Ni	0.027±0.004	5	5.4±0.019	107.46	9.81
Co	0.0136±0.001	5	5.56±0.019	110.94	7.35
