# Supplementary material for: Levels of heavy metals in soil and vegetables and associated health risks in Mojo area, Ethiopia
Source: PLoS One. 2020 Jan 30;15(1):e0227883. doi: 10.1371/journal.pone.0227883 (PMC6992214; doi:10.1371/journal.pone.0227883)
Supplement: Attachment — Submitted filename: Response to Reviewers.docx [file pone.0227883.s007.docx]

**General Remark**

First of of all we would like to thank all the Editors and reviewers of PLOS ONE for the opportunity. We really appreciate the swift response and genuine & very constructive review comments we have received on our manuscript. We have gone though our manuscript once again and tried to address all the comments as much as possible. Thank you very much once again!

**Response on the points raised by the Academic Editor**

**Journal Requirements:**

1. Regarding the formatting style of PLOS ONE, we have carefully looked at the format and corrected our manuscript accordingly.
2. The captions for supporting information have been provided at the end of the manuscript and in-text citations of the document were also carefully updated.
3. Regarding the permit for sample collection, since we have collected the samples directly from farmland (we have included the information to the methodology part under vegetable sample collection section) by purchasing from the farmers, we have not been required to obtain permission to collect the samples.
4. The map we have used in the manuscript (Fig 1) was newly made based on the GIS data we have taken during sample collection. So, we own the copy right and we do not have any objection if anyone use it in any way as long as they acknowledged us through proper citation of the article if published.
5. We have removed the financial support information from the manuscript and made necessary corrections on the funding statement section of the online submission form.

**Response to Reviewers Comment**

**Reviewer #1**

The comments forwarded are very constructive and helped us to enrich our paper.

- We have carefully checked the references and made necessary corrections in conformity with the journal format.
- The space between the paragraphs have also been checked and any irregularities were corrected.
- We have shortened the conclusion as per the suggestion.

**Reviewer #2**

We really appreciate the reviewer for the genuine and constructive comments. The comment given are very helpful and therefore, all the suggestions have been given due attention and corrections have been made accordingly.

- The title has been shortened as suggested
- The statements suggested to be removed from the abstract section have been removed accordingly.
- We have included one of the reference suggested by the reviewer while the others are some what similar with the already cited references.
- Line 115 >> we have made the suggested corrections
- The optimization of the digestion procedures have been explained and based on the suggestion given we have tried to elaborate it more. The detain procedures and the results obtained have also been explained under Results and Discussion section as well.
- In this particular study, we have focused on the adults as adults are the prominent consumer of the indicated vegetables. In principle it is necessary and again possible to calculate the EDI, THQ and TCR for children as well. However, for the sake of focus only, we have concentrated on adults.
- We have shortened the conclusion part as per the suggestion.
