# Supplementary material for: Levels of heavy metals in soil and vegetables and associated health risks in Mojo area, Ethiopia
Source: PLoS One. 2020 Jan 30;15(1):e0227883. doi: 10.1371/journal.pone.0227883 (PMC6992214; doi:10.1371/journal.pone.0227883)
Supplement: Attachment — Submitted filename: Response to Reviewers.docx [file pone.0227883.s008.docx]

**Response to Reviewers**

Reviewer #2

1. We have revisited the introduction part and included the recommended reference.
2. We checked once again the conclusion section and shortened it as much as possible as per the suggestion from the respected reviewer.
